# Negative Emotional Events that People Ruminate about Feel Closer in Time

**DOI:** 10.1371/journal.pone.0117105

**Published:** 2015-02-25

**Authors:** Ewa Siedlecka, Miriam M. Capper, Thomas F. Denson

**Affiliations:** School of Psychology, UNSW Australia, Sydney, NSW, Australia; Liaoning Normal University, CHINA

## Abstract

Rumination is intrusive, perseverative cognition. We suggest that one psychological consequence of ruminating about negative emotional events is that the events feel as though they happened metaphorically “just yesterday”. Results from three studies showed that ruminating about real world anger provocations, guilt-inducing events, and sad times in the last year made these past events feel as though they happened more recently. The relationship between rumination and reduced temporal psychological distance persisted even when controlling for when the event occurred and the emotional intensity of the event. Moreover, angry rumination was correlated with enhanced approach motivation, which mediated the rumination-distance relationship. The relationship between guilty rumination and distance was mediated by enhanced vividness. Construal level and taking a 3^rd^ person perspective contributed to the sense of distance when participants were prompted to think about less emotionally charged situations. A meta-analysis of the data showed that the relationship between rumination and reduced distance was significant and twice as large as the same relationship for neutral events. These findings have implications for understanding the role of emotional rumination on memory processes in clinical populations and people prone to rumination. This research suggests that rumination may be a critical mechanism that keeps negative events close in the heart, mind, and time.

## Introduction

When people think about a negative emotional event from their life, they often report that the event feels as though it happened “just yesterday”. At other times, people may brush off the event as happening “a long time ago” or “in a previous life”. Recent research suggests that psychological distance generally lessens the intensity of emotional experiences. However, no research has identified how cognitive-emotional processes contribute to this sense of closeness or distance. In the present research, we provide evidence that rumination maintains the feeling of temporal closeness over time. Rumination is perseverative cognition, typically about personally meaningful events that elicit negative emotions. We show that the more people dwell on negative events, the more these events feel as though they took place “just yesterday”.

Although no research has examined how rumination affects perceptions of temporal distance, there is some suggestive evidence from people who have suffered severe trauma. People with posttraumatic stress disorder (PTSD) often experience frequent and intense anger, guilt, sadness, and intrusive rumination [1]. They also experience flashbacks in which the traumatic event is relived as though it were happening in the present moment. People with PTSD often lose awareness that they are recalling a past event and show confusion about the temporal sequence of events [2]. Moreover, a meta-analysis of trauma victims found that anger was positively correlated with PTSD symptoms and this effect became stronger with increasing time since the traumatic event [3]. These findings are consistent with the possibility that ruminating about negative events makes them feel as though they took place in the recent past.

Psychological distance is the subjective feeling of distance in time, space, social distance, or likelihood of occurrence [4]. The present research focuses on perceptions of temporal psychological distance, which describes how near (or far) in the past (or future) from the present moment an event may feel. Indeed, an event that occurred years ago may paradoxically feel as though it happened more recently than an event that happened just last week. The most prominent theory of psychological distance is construal level theory [4]. The primary tenet of construal level theory is that when people think abstractly, a wide variety of physical and psychological objects seem more distant. Conversely, when people think in more concrete terms, objects seem closer in time, space, and likelihood of occurrence. Moreover, these relationships between distance and level of construal are bidirectional. Distance leads to more abstract construals and closeness to more concrete construals.

Construal level theory has provided a firm foundation for understanding the effects of psychological distance and thinking abstractly versus concretely on “cold” cognitive processes. However, relatively little is known about how “hot”, emotional processes influence perceptions of psychological distance and vice versa. A small body of research has found that psychological closeness intensifies emotional reactions, whereas distance reduces the intensity of emotional reactions and enhances emotional detachment [5–10].

This small but growing body of research suggests that construal level theory may need to be expanded to incorporate “hot” emotional processes. Perhaps the most convincing evidence to date comes from a series of 12 online studies in which psychological distance (near versus far) and construal level (abstract versus concrete) were manipulated [9]. Participants then evaluated a number of targets including going out drinking, having a hangover, going to a concert, and getting food poisoning, among others. Results showed that distance and construal level exerted independent effects on emotional intensity. Psychological distance decreased the intensity of both positive and negative emotional reactions, whereas abstract thinking increased positive feelings toward the targets of evaluation. Moreover, distance decreased emotional intensity even when participants were prompted to think about the very best and very worst things that might happen to them. Another series of studies found that writing about events in emotional terms versus neutral terms decreased psychological distance [10]. The events included embarrassments, trips to the dentist, dreaded or desired upcoming events, and the 2007 Virginia Tech shootings. Another research program has examined how self-distancing can lower the intensity of negative emotions and reduce rumination [7, 11 – 12]; In sum, distancing reduces the intensity of emotional experiences and may reduce rumination, yet it remains unknown what effect rumination has on feelings of temporal distance.

These findings are promising, but this past research did not examine specific emotions and leaves open the possibility that other factors may contribute to a sense of closeness versus distance. The present research addressed this gap by testing the role of rumination about three specific emotions (anger, guilt, and sadness) in inducing feelings of temporal closeness. Rumination often occurs in response to a personally meaningful negative event and represents a form of processing whereby people mentally replay events in an effort to induce closure or increase understanding of the causes and consequences of the event [13]. Moreover, people tend to believe that rumination is an effective way to cognitively process events that induce anger or depression [14–16]. Thus, we suggest that rumination may represent a form of motivated cognition whereby people are inclined to keep negative events close at hand for which closure or greater understanding is desired.

### Overview of the Present Research

In three studies we discovered that rumination about real world autobiographical events that induced anger, guilt, and sadness was positively related to perceptions of temporal closeness. In Study 3, we examined alternative explanations and mediators for the reduced temporal distance induced by rumination. We found that rumination was the strongest predictor of reduced temporal distance for the three emotions. Moreover, enhanced approach motivation and vividness mediated the effects of angry and guilty rumination on reduced distance, respectively. However, for more affectively neutral events (i.e., “cold” cognitive processing), variables derived from construal level theory (i.e., abstract thinking and taking a third-person perspective) predicted greater temporal psychological distance.

### Ethics Statement

The UNSW Australia Human Research Ethics Committee approved all research, which was conducted in accordance with the Declaration of Helsinki. All participants provided informed consent. Specifically, at the bottom of the consent form, the following statement appeared: “To indicate your consent to the terms outlined above, please mark one of the following.” Participants were given two options; they either clicked “I accept” and entered their initials or clicked “I do not accept”. Only participants who clicked “I accept” were allowed to begin the study. This consent procedure was approved by the UNSW Australia Human Research Ethics Committee. On average, participants took 5–10 minutes to complete each study.

## Study 1

In Study 1, we examined the relationship between angry and guilty rumination about autobiographical experiences and perceptions of temporal psychological distance. Because rumination occurs over time following negative events, we asked people to recall a time when they felt angry, guilty, or a trip to the supermarket during the past year (i.e., a relatively emotionally neutral event). Just as people may dwell on times when they are harmed (angry rumination), people are also likely to ruminate when they harm others (guilty rumination). We then enquired how often they had thought about the event, their emotional responses, and perceived temporal distance. We hypothesized that rumination would be related to a sense of temporal closeness above and beyond when the event actually occurred and the emotional intensity of the event.

### Method

Participants and Design

Our aim was to obtain a sample of 100 per group. A total of 306 Americans participated in the online research via Amazon’s Mechanical Turk in exchange for US$0.50. Data from 18 participants were excluded based on incorrectly answering an attention check (*n* = 6) or not following instructions (*n* = 12). Specifically, at the end of Studies 1–3, participants completed the Ten-item Personality Inventory [17]. The purpose was to include an attention check. We included an item for which the instructions were to “please click 6”. Participants who responded incorrectly were eliminated from analyses. The remaining sample was 288 participants (117 women; *M*_*age*_ = 29.51 years, *SD*_*age*_ = 10.34; 72% Caucasian, 15% Asian). Based on their month of birth, participants were assigned to recall an anger-inducing event (*n* = 91), a guilt-inducing event (*n* = 97), or a typical trip to the supermarket (*n* = 100). Men and women were equally distributed among the groups, χ^2^(2, *N* = 288) = 3.75, *p* = .15.

Event Recall

Participants were asked to briefly describe a personal event that occurred within the past year that induced anger, guilt, or a neutral trip to the supermarket.

Emotional Responses to the Event

After describing the event, participants completed a modified version of the Positive and Negative Affect Schedule (PANAS) to report on how they felt at the time of the event [18]. The scale consisted of 30 emotional descriptors measuring angry affect (i.e., angry, furious, irritable, hostile, enraged, agitated, and mad; α = .97), positive emotions (e.g., pleased; α = .93), and other negative emotions (e.g., afraid; α = .89). The items *guilty* and *ashamed* were used to examine guilty affect (α = .91) (1 = very slightly or not at all; 5 = extremely). Participants also rated the overall intensity of their emotional response to the event (1 = not intense at all; 7 = very intense).

Temporal Psychological Distance

We computed perceived temporal distance from three items: “How close or far away in time does the event feel?” (1 = feels very close to 7 = feels very far away); “How long ago in time does the event seem to you?” (1 = feels very recent to 7 = feels very long ago); “How near or distant in time does the event feel?” (1 = feels very near to 7 = feels very distant) (α = .95) Participants then reported how many months ago the event actually occurred.

Rumination

We contained a single item measure of rumination from our prior work [19 – 20]. Specifically, participants were asked to rate how often they thought about the event within the past year (1 = not at all; 7 = very often).

### Results and Discussion

Preliminary Analyses

As shown in Table 1, we observed differences on angry affect and guilty affect depending on which event participants recalled. Planned contrasts showed that participants who recalled an anger-inducing event reported more angry affect than participants in the remaining two groups, *t*(285) = 18.93, *p* < .0001. Likewise, people who recalled a guilt-inducing event reported more guilty affect than participants in the remaining two groups, *t*(285) = 23.47, *p* < .0001. Planned contrasts also showed that participants who recalled anger and guilt-inducing events reported more intense emotional responses to the events than those in the supermarket group, *t*(285) = 13.71, *p* < .0001. Participants who recalled an anger-inducing event also reported a more intense emotional response than participants who recalled a guilt-inducing event, *t*(285) = 2.35, *p* = .02. Further contrasts showed that participants in the emotion groups reported thinking about the event more during the past year than participants who recalled the supermarket experience, *t*(285) = 12.17, *p* <.0001. The anger and guilt groups did not differ on rumination, *t*<1.

**Table 1:** Means and (standard deviations) of the measures from Studies 1 and 2.

STUDY 1								
	Anger		Guilt		Control		
	(*n* = 91)		(*n* = 97)		(*n* = 100)		
	*M*	*SD*	*M*	*SD*	*M*	*SD*	*F*(2,285)	η^2^
Angry affect	3.59_a_	1.03	1.99_b_	0.98	1.17_c_	0.38	203.98***	0.59
Guilty affect	1.65_a_	0.97	3.90_b_	1.01	1.15_c_	0.5	286.73***	0.67
Emotional intensity	5.46_a_	1.5	4.93_b_	1.61	2.55_c_	1.56	96.19***	0.4
Rumination	4.59_a_	1.69	4.71_a_	1.75	2.15_b_	1.55	74.28***	0.34
Months ago event occurred	4.03_a_	2.92	4.27_a_	3.13	0.96_b_	0.97	52.88***	0.27
Temporal distance	2.71_a_	1.64	2.55_a_	1.63	2.13_b_	1.08	4.07*	0.03

STUDY 2								
	Anger		Guilt		Control			
	(*n* = 102)		(*n* = 80)		(*n* = 96)			
	*M*	*SD*	*M*	*SD*	*M*	*SD*	*F*(2,275)	η^2^
Angry affect	3.55_a_	1	2.12_b_	0.95	1.23_c_	0.55	187.74***	0.58
Guilty affect	1.58_a_	0.82	3.81_b_	1.04	1.19_c_	0.54	258.33***	0.65
Emotional intensity	5.26_a_	1.46	4.90_a_	1.76	3.54_b_	1.6	31.19***	0.19
Rumination	1.92_a_	0.65	2.08_a_	0.73	1.50_b_	0.57	19.55***	0.12
Months ago event occurred	4.46_a_	3.38	5.14_a_	3.33	5.18_a_	3.28	1.42	0.01
Temporal distance	2.91_a_	1.53	2.99_a_	1.66	3.54_b_	1.56	4.56*	0.03

Within a row, means that with different subscripts are significantly different (*p* < .05).

There was an unexpected difference between the groups on how many months ago the event occurred. Participants in the supermarket group described a more recent event than participants who recalled anger and guilt-inducing events, *t*(285) = -10.25, *p* < .0001. Therefore, there was a parallel pattern of results for perceived temporal distance. Participants in the supermarket group reported closer temporal distance than participants in the anger and guilt groups, *t*(285) = -2.78, *p* = .006. Moreover, months since the event occurred and perceived distance were significantly correlated, *r*(286) = .40, *p* < .0001. We addressed these limitations in Study 2. Nonetheless, because our primary hypothesis was that rumination would be related to reduced temporal psychological distance, we conducted further analyses controlling for how many months ago the event actually occurred. When controlling for when the event occurred, overall emotional intensity was also correlated with perceived distance and was therefore included as a covariate, *r*_*partial*_(285) = -.16, *p* = .006.

Temporal Psychological Distance

We conducted a regression analysis to test the extent to which angry and guilty rumination would affect perceived temporal distance. As seen in Table 2, rumination was inversely related to perceived distance for participants who recalled anger and guilt-inducing events. The rumination-distance relationship was not significant for participants who recalled a trip to the supermarket. In sum, Study 1 found that the more people reported thinking about an anger-inducing provocation or a guilt-inducing event during the past year, the more recent the negative event felt in time. This effect occurred even when controlling for when the event actually occurred and emotional intensity.

**Table 2:** Regression analyses from Study 1 predicting temporal psychological distance.

	Whole sample		Anger		Guilt		Control	
	(*N* = 288)		(*n* = 91)		(*n* = 97)		(*n* = 100)	
	*R* ^*2*^ = .25***		*R* ^*2*^ = .33***		*R* ^*2*^ = .23***		*R* ^*2*^ = .14**	
	β	*t-value*	β	*t-value*	β	*t-value*	β	*t-value*
Rumination	-.34***	-4.64	-.46***	-4.64	-.21*	-2.1	-0.09	-0.83
Emotional intensity	-0.03	-0.4	0.06	0.65	-0.06	-0.54	-0.12	0.81
Months ago event occurred	.45***	7.47	.25***	4.96	.41***	4.46	.31**	3.25

The left column represents the whole sample controlling for condition. Regression coefficients are standardized.

***p < .001.

** *p* < .01.

*p < .05.

## Study 2

The aim of Study 2 was to replicate the findings of Study 1 while simultaneously addressing two limitations. First, due to our use of a trip to the supermarket as a neutral topic in Study 1, participants reported a more recent event than participants who recalled an anger or guilt inducing event (presumably because people go grocery shopping more often than they experience high levels of anger or guilt). We therefore altered our neutral topic in Study 2 to equate the groups on time since the event actually occurred. Second, in Study 2 we used a more conventional measure of rumination.

### Method

Participants and Design

Our aim was to obtain a sample of 100 per group. A total of 303 Americans participated in the online research via Amazon’s Mechanical Turk in exchange for US$0.50. Data from 25 participants were excluded based on incorrectly answering the attention check (*n* = 6) or failure to follow instructions (*n* = 19). The remaining sample was 278 participants (101 women; *M*_*age*_ = 28.28 years, *SD*_*age*_ = 9.04; 70% Caucasian, 16% Asian). Based on their date of birth, participants were asked to recall an anger-inducing event (*n* = 102), a guilt-inducing event (*n* = 80), or an ordinary social interaction (*n* = 96). Men and women were equally distributed among the groups, χ^2^(2, *N* = 278) = 0.65, *p* = .72.

Event Recall

Participants were asked to briefly describe a personal interaction that occurred within the past year that induced anger, guilt, or a neutral event. Because participants in Study 1 in the neutral group recalled very recent trips to the supermarket, the neutral topic was changed to describing “…an ordinary interaction with someone when you were out of town.” This change was successful in equating the groups on actual temporal distance from the event, *F*(2,275) = 1.42, *p* = .24, η^2^ = .01 (Table 1).

Emotional Responses to the Event

Participants completed the same PANAS as in Study 1 assessing angry affect (α = .96), positive emotions (α = .94), other negative emotions (α = .85), and guilty affect (α = .89). Participants also rated the intensity of their overall emotional response.

Psychological Temporal Distance

Participants then completed the same three temporal distance items from Study 1 (α = .94) and reported how many months ago the event actually occurred.

Rumination

Participants completed a modified version of the Impact of Events Scale (IES) [21]. Our modified scale consisted of 8 items assessing intrusive, ruminative thoughts (e.g., “I thought about it when I didn’t mean to”; α = .87; see S1 Materials for the full measure and correlations with other study variables). Participants endorsed the frequency of the statements in the past seven days regarding the event described (1 = not at all; 4 = often).

### Results and Discussion

Preliminary Analyses

As expected, there was a difference between the recall groups on angry affect and guilty affect (see Table 1). Planned contrasts showed that participants who wrote about the anger-inducing event reported more angry affect than participants in the remaining two groups, *t*(275) = 17.71, *p* < .0001. Likewise, people who wrote about the guilt-inducing event reported more guilty affect than participants in the remaining two groups, *t*(275) = 22.53, *p* < .0001. A planned contrast also showed that participants who wrote about anger and guilt-inducing events reported more intense emotional responses to the events than participants who wrote about an ordinary social interaction, *t*(275) = 7.62, *p* < .0001. Participants who wrote about anger and guilt-inducing events also reported feeling equivalent levels of overall emotional intensity, *t*(275) = 1.53, *p* = .13. Participants in the emotion recall groups reported more intrusive and perseverative thoughts about the event during the past seven days than participants who wrote about an ordinary social interaction, *t*(275) = 6.12, *p* < .0001. Participants who wrote about anger and guilt-inducing events reported equivalent levels of rumination, although there was a trend for more rumination in the guilt group than in the anger group, *t*(275) = 1.71, *p* = .089.

Temporal Psychological Distance

**Fig 1:**
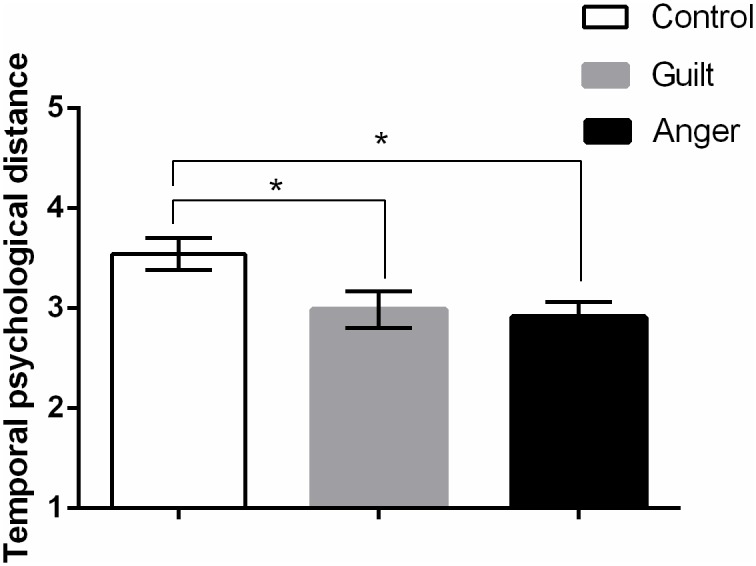
Means and SEMs for perceptions of psychological distance in Study 2 (*N* = 278). Higher scores indicate greater temporal psychological distance. Participants who wrote about anger- or guilt-inducing transgressions perceived the event as being closer in time than participants who wrote about a neutral event. The two emotion conditions did not differ in perceived psychological distance. * *p* < .05.

Participants who wrote about an anger-inducing event, *t*(275) = -2.81, *p* = .005, or a guilt-inducing event reported closer temporal distance than participants who wrote about a normal social interaction, *t*(275) = -2.32, *p* = .021 (Table 1 and Fig. 1).

Table 3 shows the results of the four regression analyses that controlled for when the event occurred (in months) because it was correlated with perceived distance, *r*(276) = .41, *p* < .001. Emotional intensity was also correlated with temporal distance and therefore included as a covariate, *r*_partial_(275) = -.28, *p* < .001. As in Study 1, we tested the hypothesis that rumination would be inversely related to perceived distance for participants who recalled anger and guilt-inducing events. As shown in Table 3, this was indeed the case. Rumination was negatively related to distance in the anger group and marginally in the guilt group (*p* = .068), but not the neutral group. In sum, Study 2 replicated the finding of Study 1: the more participants reported ruminating about negative events, the closer in the past the event felt.

**Table 3:** Regression analyses from Study 2 predicting temporal psychological distance.

	Whole sample		Anger		Guilt		Control	
	(*N* = 278)		(*n* = 102)		(*n* = 80)		(*n* = 96)	
	*R* ^*2*^ = .25***		*R* ^*2*^ = .25***		*R* ^*2*^ = .26***		*R* ^*2*^ = .21***	
	β	*t-value*	β	*t-value*	β	*t-value*	β	*t-value*
Rumination	-.15*	-2.48	-.22*	-2.32	-.21ǂ	-1.86	-0.01	-0.06
Emotional intensity	-.17**	-2.63	-.18ǂ	1.84	-0.15	-1.23	-0.14	-1.5
Months ago event occurred	.43***	7.96	.37***	4.1	.51***	4.8	.44***	4.75

The left column represents the whole sample controlling for condition. Regression coefficients are standardized.

***p < .001.

** *p* < .01.

*p < .05.

ǂ *p* < .10.

## Study 3

The aim of Study 3 was to test the extent to which rumination would influence perceptions of temporal psychological distance above and beyond variables known to influence psychological distance derived from construal level theory. For “cold” cognitive processes, psychological closeness is associated with low-level construals, ease of retrieval (i.e., fluency), taking a first-person perspective, and focusing on the specific details of an event [4]. Thus, we examined the potential effects of perceptual fluency, construal level, mental representation of the event and visual perspective on perceptions of distance [10]. We also examined the role of vividness because there is evidence that the vividness of mental imagery may mediate the effects of (albeit positive) emotions on psychological distance [22]. Finally, we also examined whether self-reported approach motivation would predict additional variance in temporal distance. Our motivated cognition perspective suggests that angry rumination maintains approach motivation to exact revenge (angry rumination) [23]. Similarly, guilty rumination may maintain approach motivation to repair harm done [24]. We also included sadness because it is generally considered to be an avoidance-oriented emotion, whereas anger and guilt are approach-oriented emotions [24 – 25]. Thus, we expected approach motivation to explain the variance in angry and guilty rumination, but not depressive rumination. In general, we also expected that rumination would be a key predictor of psychological closeness for the emotional events, but not the control event. By contrast, we expected to replicate prior work showing that the variables derived from construal level theory would better predict distance for people who recalled the less emotional “cold”, control event.

### Method

Participants and Design

Because we investigated more variables than in the first two studies, our aim was to double the sample size by recruiting at least 200 participants per group. A total of 1,110 Americans participated in an online study via Amazon's Mechanical Turk in exchange for US$1.00. Data from 283 participants were excluded based on incorrectly answering an attention check (*n* = 145) or not following instructions (*n* = 202) (e.g., writing about a different topic than that assigned to them). The remaining sample was 827 participants (369 women; *M*_*age*_ = 31.63 years; *SD*_*age*_ = 10.74; 77.8% Caucasian, 7.0% African American). Participants were randomly assigned to write about a time in which they felt angry (*n* = 191), guilty (*n* = 215), sad (*n* = 209), or a control topic (*n* = 212). Men and women were equally distributed among the conditions, χ^2^(3, *N* = 826) = 3.87, *p* = .28.

Event Recall

The anger, guilt, and control event were identical to Study 2. For the sad event, participants were asked to write about a time in which they felt sad within the past year.

Emotional Responses to the Event

Participants completed the same PANAS as in Studies 1 and 2 assessing angry affect (α = .96), positive emotions (α = .94), other negative emotions (α = .82), and guilty affect (α = .87). Participants also rated the intensity of their overall emotional response.

Rumination

Participants completed the same modified version of the Impacts of Events Scale as in Study 2 (α = .89).

Temporal Psychological Distance

Participants completed the same temporal psychological distance items from Studies 1 and 2 (α = .96) and how many months ago the event actually occurred.

Control Variables and Mediators

We adopted most of our control variables from van Boven et al. [10]. Perceptual fluency was assessed by asking participants to report how easy or difficult it was to recall the anger-inducing event (1 = very difficult, 7 = very easy). Following van Boven et al. [10], the concepts of construal level and concreteness were explained to participants. Two items then assessed how participants thought about the anger-inducing event in terms of high and low levels construals (1 = low-level construal, 7 = high-level construal) and how they mentally represented the event (1 = specific details/how the event happened, 7 = broader meaning/why the event happened). The two construal level variables were significantly correlated and therefore averaged into a single item (*r* = .50, *p* < .001). The role of visual perspective was examined by asking participants to report the extent to which they thought about the event from a first or third person perspective (1 = my own eyes, 7 = an observer’s eyes). Vividness was examined by asking participants to report how vividly they could recall the anger-inducing event (1 = very vividly, 7 = not at all vividly). Depending on which event participants recalled, approach motivation was assessed by asking people “In regards to the interaction you described, to what extent would you like to approach the person who made you angry (anger-inducing event); take action to eliminate guilty feelings (guilt-inducing event); approach the person who made you sad (sadness-inducing event); or approach the person whom you met while out of town?” (neutral event) (1 = not at all; 7 = very much).

### Results and Discussion

Preliminary Analyses

The data are presented in Table 4. Planned contrasts showed that participants who wrote about the anger-inducing event reported more angry affect than participants in the remaining three groups, *t*(823) = 19.51, *p* < .0001. Likewise, participants who wrote about the guilt-inducing event reported more guilty affect than participants in the remaining three groups, *t*(823) = 29.08, *p* < .0001, and people who wrote about the sadness-inducing event reported more negative affect than people in the remaining three groups, *t*(823) = 3.24, *p* = .001. Further planned contrasts showed that participants in the three emotion groups reported more intense emotional responses to the events than those who wrote about the ordinary social interaction, *t*(823) = 14.22, *p* < .0001. Similarly, participants in the emotion groups reported more intrusive and perseverative thoughts about the event during the past seven days than participants who wrote about an ordinary social interaction, *t*(823) = 13.94, *p* < .0001. Participants who recalled emotion-inducing events also reported adopting a more abstract construal level than participants who wrote about an ordinary social interaction, *t*(823) = 8.29, *p* < .0001. There were also significant ANOVA results for approach motivation and visual perspective, but the post hoc tests were not significant. The type of event recalled did not influence fluency or vividness.

**Table 4:** Means and (standard deviations) of the measures from Study 3.

	Anger		Guilt		Sadness		Control		
	(*n* = 191)		(*n* = 215)		(*n* = 209)		(*n* = 212)		
	*M*	*SD*	*M*	*SD*	*M*	*SD*	*M*	*SD*	*F*(3,823)	η^2^
Angry affect	3.39_a_	1.12	1.87_b_	0.98	2.35_c_	1.19	1.22_d_	0.52	174.19***	0.39
Guilty affect	1.53_a_	0.82	3.59_b_	1.17	1.90_c_	1.02	1.12_d_	0.38	308.93***	0.53
Negative affect	1.98_a_	0.69	2.56_a_	0.86	2.22_a_	0.79	2.01_b_	0.86	69.76***	0.2
Emotional intensity	5.43_a_	1.25	4.57_b_	1.52	5.19_a_	1.38	3.43_c_	1.58	79.07***	0.22
Rumination	1.92_a_	0.67	1.89_a_	0.69	2.06_a_	0.72	1.24_b_	0.45	67.71***	0.2
Months ago event actually occurred	4.43_a_	3.55	4.88_a,b_	3.88	5.07_a,b_	3.78	5.62_b_	3.74	3.53*	0.01
Temporal distance	3.40_a_	1.67	3.52_a_	1.68	3.45_a_	1.79	4.09_b_	1.6	7.46***	0.03
Approach motivation	4.26_a_	2.05	4.71_a_	1.77	4.47_a_	2.17	4.77_a_	1.93	3.15*	0.01
Fluency	5.87	1.4	5.77	1.47	5.63	1.57	5.95	1.37	1.88	0.01
Construal level	4.06_a_	1.54	4.27_a_	1.53	4.53_b_	1.35	3.30_c_	1.56	26.52***	0.09
Visual perspective	2.45_a_	2.24	3.05_a_	2.58	3.09_a_	2.54	3.11_a_	2.83	3.00*	0.01
Vividness	5.5	1.48	5.38	1.54	5.35	1.41	5.28	1.44	0.8	0

Within a row, means that end with different subscripts are significantly different (Scheffe’s *p* < .05).

***p < .001.

** *p* < .01.

*p < .05.

Temporal Psychological Distance

**Fig 2:**
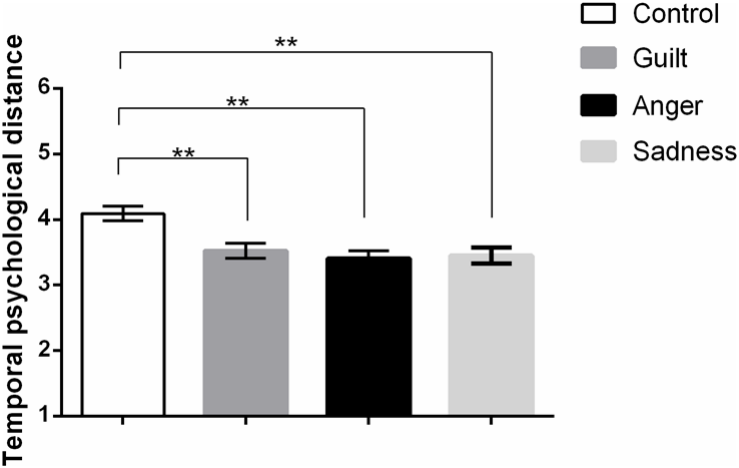
Means and SEMs for perceptions of temporal psychological distance in Study 3 (*N* = 827). Higher scores indicate greater temporal psychological distance. Participants who wrote about anger-, sadness- or guilt-inducing events perceived the event as closer in time than participants who wrote about a neutral event. The three emotion conditions did not differ in perceived psychological distance. ** *p* < .01.

The temporal distance data are presented in Table 4 and Fig. 2. A planned contrast showed that participants who recalled emotional events reported closer temporal distance than participants who recalled the control topic, *t*(823) = -4.69, *p* < .0001. Post hoc tests of each emotion event group versus the control group showed that participants who wrote about emotion-inducing events reported feeling as though the event happened more recently than participants who wrote about the control topic, all pairwise *p*s < .008.

The multiple regression analyses for each emotion condition and the whole sample are presented in Table 5. There was a small inverse relationship between rumination and distance in the control group. This correlation was driven by a minority of participants who reported ruminating about their interaction while out of town. Over 60% of participants in the control group reported no rumination whatsoever in the past 7 days compared to 13%, 16% and 12% in the anger, guilt, and sad events groups, respectively. Moreover, the magnitude of the relationship between rumination and distance was at least twice as large in each of the emotion-inducing groups as that in the control group. Indeed, rumination was the strongest predictor of distance for participants who recalled emotion-inducing events, but not for participants who recalled the control topic.

**Table 5:** Regression analyses from Study 3 predicting temporal psychological distance.

	Whole sample		Anger		Guilt		Sadness		Control	
	(*N* = 827)		(*n* = 191)		(*n* = 215)		(*n* = 209)		(*n* = 212)	
	*R* ^*2*^ = .24***		*R* ^*2*^ = .26***		*R* ^*2*^ = .22***		*R* ^*2*^ = .28***		*R* ^*2*^ = .23***	
	β	*t-value*	β	*t-value*	β	*t-value*	β	*t-value*	β	*t-value*
Rumination	-.38***	-10.15	-.36***	-5.1	-.36***	-5.18	-.44***	-6.44	-.18**	-2.65
Emotional intensity	-0.04	-0.91	0.02	0.22	-0.03	-0.43	0.06	0.81	-.22**	-2.83
Months ago event occurred	.16***	5.31	.21**	3.23	0.03	0.47	.18**	2.88	.22***	3.57
Approach motivation	-.11**	-3.46	-.23 **	-3.43	-0.06	-0.82	-0.02	-0.32	-.13ǂ	-1.83
Fluency	-0.04	-1.25	-0.09	-1.22	-0.03	-0.39	-0.05	-0.74	0.02	0.22
Construal level	.09**	2.6	0.07	1	0	0.03	0.06	0.94	.21**	3.11
Visual perspective	.06ǂ	1.86	0.05	0.73	-0.01	-0.15	0.03	0.4	.13*	1.97
Vividness	-.11**	-3.15	-0.1	-1.34	-.15 *	-2.25	-.12ǂ	-1.79	-0.05	-0.7

The left column represents the whole sample controlling for condition. Regression coefficients are standardized. Underlined coefficients indicate significant mediators.

***p < .001.

** *p* < .01.

*p < .05.

ǂ *p* < .10.

As expected, approach motivation was also inversely related to perceived distance for participants who recalled the anger-inducing event, but contrary to our hypothesis, not for participants who recalled the guilt-inducing event. Vividness significantly predicted lower perceptions of distance for participants who recalled the guilt-inducing event and marginally so for participants who recalled the sadness-inducing event. By contrast, for participants who wrote about an ordinary social interaction, there were three additional variables that significantly contributed to perceived distance. These included emotional intensity, construal level, and visual perspective. These findings suggest that rumination about negative emotional events is the strongest correlate of decreased perceived temporal distance. By contrast, in less emotionally charged situations, construal level and visual perspective are associated with perceived distance.

Mediation Analyses

We conducted mediation analyses within each emotion condition to identify mediators of the rumination-distance link. We used Hayes’ [26] PROCESS Model 4 script for SPSS with 50,000 resamples. All variables were entered as potential mediators and we controlled for when the event occurred. Approach motivation significantly mediated the rumination-distance relationship for participants who recalled the anger-inducing event as indicated by a 95% confidence interval (CI) for an indirect effect estimate (-0.088, *SE* = .053) that did not cross zero (CI = -.225,-.011). These data suggest that angry rumination contributes to closeness by maintaining approach motivation. For participants who recalled the guilt-inducing event, vividness significantly mediated the effect of rumination on perceived distance as indicated by a 95% confidence interval (CI) for an indirect effect estimate (-0.075, *SE* = .046) that did not cross zero (CI = -.196,-.009). There were no mediators for participants who recalled the sadness-inducing event and we did not conduct mediation analyses in the control condition due to overall low levels of rumination. The mediation analyses suggest that angry rumination enhances approach motivation which in turn makes the provocation feel as though it happened more recently. Guilty rumination enhances the vividness of the event, which decreases perceived temporal distance.

In sum, Study 3 replicated and extended the main findings of Studies 1 and 2 showing that negative emotional events that people ruminate about seem as though they happened more recently. Moreover, approach motivation and vividness associated with angry and guilty rumination, respectively, contributed to the sense of closeness in time. Construal level and vantage perspective contributed to the sense of closeness for a less emotionally charged event (i.e., a normal interaction with someone while out of town).

## Meta-Analysis

**Fig 3:**
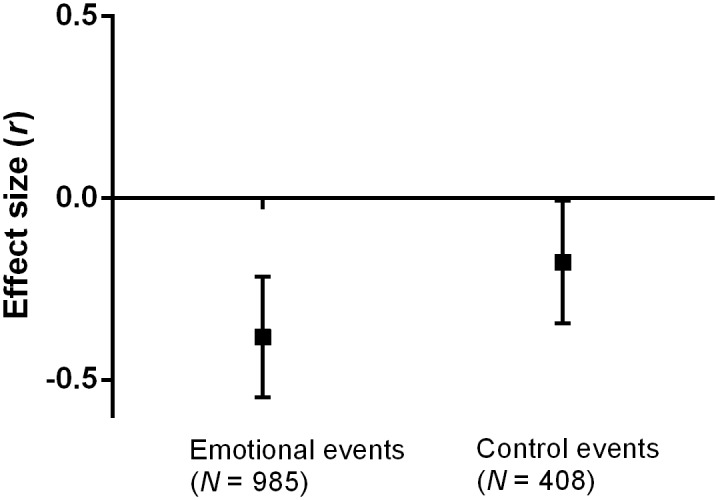
Meta-analysis of the effect of rumination on reduced temporal distance from Studies 1 through 3. Values represent the mean effect sizes and 95% confidence intervals (i.e., the mean ± 1.96 SEMs). The effect size was significantly different and more than twice as large for rumination about emotional events compared to thinking about control events.

We conducted a meta-analysis of the association between rumination and temporal psychological distance from the data reported in Studies 1–3. Following procedures outlined by Wilson and Lipsey [27], we first calculated the partial correlation between rumination and distance for each condition, then used Fisher’s *r*-to-Z transformation and weighted each effect size by the inverse variance. We then calculated mean effect sizes collapsed across all of the emotion recall groups versus the control groups. For ease of interpretation we converted effect sizes back to *r* and calculated 95% CIs (Fig. 3). This meta-analysis showed a significant, medium-to-large association between rumination and reduced temporal distance for the emotional events, *r* = -.392 (95% CI = -.242,-.524). There was also a smaller, but significant relationship between rumination and reduced distance in the control groups as indicated by a 95% CI that just missed zero, *r* = -.176 (95% CI = -.008,-.335). Nonetheless, the difference in effect sizes was significantly larger for emotional events than the control events, *Z*(*N*_*emotion*_ = 985; *N*_*control*_ = 408) = 4.00, *p* < .001. In sum, emotionally negative events that people ruminate about feel as though they happened more recently to a greater extent than thinking about less emotional events.

## General Discussion

The present research contributes to the literature by identifying rumination about negative events as a correlate of reduced temporal psychological distance. This reduced distance occurred for a range of negative emotional events that induced rumination. Moreover, a meta-analysis of the data showed that the relationship between rumination on reduced perceptions of temporal distance was greater for negative emotional events than more neutral events. This effect of rumination occurred over and above the emotional intensity of the event, construal level, perceptual fluency, vantage perspective, and when the event actually occurred. Moreover, Study 3 identified enhanced approach motivation and vividness as partial meditators of the rumination-distance relationship for anger- and guilt-inducing events, respectively. In sum, we replicated past work showing that construal level influences perceived distance for relatively “cold” cognitive processing and emotional intensity reduces perceptions of distance for emotional events [4, 10]. However, the unique contribution of the current research was to identify rumination as a relatively powerful correlate of reduced distance above and beyond intensity and construal level. Thus, emotional intensity may initially contribute to the sense of temporal closeness, but rumination over time maintains the feeling that the negative event happened “just yesterday”.

### Implications

We examined the effects of three specific emotions on perceived temporal distance that varied in approach-avoidance motivation and are either considered basic or self-conscious. Some theorists have suggested that specific emotions may exert varying effects on perceived psychological distance, depending on whether the emotion stimulates abstract or concrete processing [4]. For instance, social emotions such as guilt may elicit a high-level construal because guilt requires taking a socially distant perspective [4, 28]. Our research did not support this notion. We found that an approach-oriented basic emotion (anger), an approach-oriented self-conscious emotion (guilt), and an avoidance-oriented basic emotion (sadness) all induced a sense of closeness. Moreover, rumination about these emotional experiences maintained that sense of closeness over time. Thus, emotional intensity may reduce perceptions of distance regardless of the specific negative emotion, but rumination is likely what makes people keep emotionally painful experiences close to their hearts and minds. Future research with a broader variety of emotion inductions, including positive emotions, will help clarify their effects on psychological distance. Indeed, it would be premature to conclude that perseverative thinking is associated with reduced distance *only* when thinking about negative events. Future research may find that perseverative thinking about positive life events similarly reduces perceived distance.

We proposed elsewhere that anger and angry rumination may heighten aggressive retaliation by keeping past provocations cognitively salient and maintaining approach motivation and physiological responses in a vigilant state of readiness [23]. In this way, anger and angry rumination about a past transgression may keep attention focused on a potential threat. Thus, perceiving a provoking event as closer in the past may be a form of motivated cognition that keeps people vigilant toward potential enemies [29]. Consistent with this notion, increased approach motivation mediated the effect of angry rumination on reduced distance. This mediating effect of approach motivation is consistent with the notion that this type of rumination focuses and maintains attention on threats to one’s safety. Indeed, others have argued that people equate closeness with danger and distance with safety [30]. In this sense, our findings are quite consistent with Xiao and van Bavel’s [29] findings that people underestimated the physical closeness of threatening outgroups. Thus, the increased closeness induced by angry rumination may be a form of motivated cognition that enhances vigilance to would-be harm doers when an opportunity for vengeance arises.

At first glance, however, the heightened approach motivation induced by angry rumination may seem at odds with research suggesting that rumination is an avoidance-related process [31]. However, our findings are quite consistent with Kelley et al. [31]. They suggest that when people are ruminating (usually not in the presence of the provocateur) they are inhibiting aggressive action, but when the opportunity for vengeance arises, angry rumination may facilitate approach-related attack. We found that one consequence of this angry rumination is a conscious awareness of the desire to approach the provocateur and this desire predicted a reduced sense of temporal distance.

We also hypothesized that approach motivation should mediate the relationship between guilty rumination and reduced distance. We did not find support for this hypothesis. In retrospect, our instructions relied on lay interpretations of the meaning of guilt. Scientific perspectives differentiate guilt from shame, often in terms of antecedent appraisals and approach-avoidance orientation. Guilt is elicited in response to the sense of doing a bad thing, which elicits approach motivation to repair the wrongdoing. Shame is elicited by appraisals of being a bad person and elicits motivation to avoid people who may elicit censure. However, in the general public, guilt and shame often become blended making the unique effects difficult to disambiguate [24]. Thus, the approach motivation induced by guilt and the avoidance motivation induced by shame may have negated each other. In order to test this possibility, in a post-hoc analysis, we examined whether participants who were asked to write about guilt wrote about shame experiences instead. Specifically, we coded the written descriptions of the events as shame-inducing, guilt-inducing or some of both. In Study 1, 94% of participants described a guilt-inducing event; in Study 2, this value was 95%; in Study 3, it was 89%. Due to the small proportion of participants who wrote about shame or a mixed experience in each study, we did not conduct separate mediation analyses on approach motivation for the subgroups of participants. Nonetheless, future research could more carefully elicit guilt versus shame.

Vividness mediated the effect of guilty rumination on reduced distance. The effects of vividness on reduced distance were smaller than those of rumination for the emotional events, but consistent across the three types of emotions (*β*s between-.10 and-.15). Thus, rumination may contribute to reduced distance via reliving the events more vividly. Indeed, people may use vividness as a heuristic cue to gauge temporal closeness. Events that are recalled most vividly are often those that happened most recently because memory is typically good for recent events (and is even better for stressful events). Thus, another contribution of the present research is to extend Alter and Balcetis’ [22] finding of a mediating effect of vividness to a negative emotion (i.e., guilt).

Frequent experiences of highly intense negative emotions and rumination are common in clinical populations such as people with PTSD, depression, and anxiety [32 – 33]. The present research may eventually be applied to strategies targeting harmful cognitive processes in these people. Anger and angry rumination may make provocations feel closer in time, but they are also likely to induce a biased narrative of the actual events [34]. By embellishing the unjustified harm done to oneself over time, angry rumination may lead to self-serving accounts of the provocation. Ceasing angry rumination may enhance feelings of temporal distance and make forgiveness possible.

People often ruminate about events for which they need not experience guilt. A classic example is “survivor guilt” for which victims of trauma feel guilty about surviving (e.g., as in a car crash or combat) while others have died [35]. In such instances, guilty rumination may keep the traumatic event highly salient in memory and thereby impede recovery. Similarly, depression is characterized by rumination about guilt as well as sadness and associated with a variety of adverse consequences. Thus, ceasing rumination could facilitate more active involvement in therapy.

### Limitations and Future Research

The present research was limited in some aspects. Although we favor a causal interpretation of rumination in determining enhanced psychological closeness, it is also possible that the effects are bidirectional (as are many effects within construal level theory). That is, people may ruminate about negative events because they feel closer in time. Future research using prospective or experimental designs could untangle these effects. Within-participants designs could also provide more precise control over individual differences in emotional recall processing. Another limitation is that we examined only three negative emotions to gauge the effects of rumination on perceived temporal distance. We selected these emotions because angry, guilty, and depressive rumination are common. Nonetheless, the current research did not include any positive emotions (e.g., gratitude for a past act of kindness). However, individuals are more inclined to ruminate about negative experiences than positive experiences [36]. The present study only considered *past* temporal psychological distance, and additionally did not consider other dimensions of psychological distance (i.e., future temporal distance, spatial, social distance, likelihood). For instance, the future feels closer to people than the past, and this sense of closeness is thought to prepare people to take impending action [37]. Similarly, students who were induced to feel that an upcoming exam was closer in time versus more distant were more motivated to achieve academic success [38]. Our findings suggest a similar effect for angry rumination. By maintaining focus on a prior provocation in the past, angry rumination is thought to motivate revenge in the future when it is relatively safe to do so [23]. Thus, one implication of the present research is that worry should induce a sense of closeness. Worry is a future-oriented form of repetitive thinking in which the focus is on an impending threat [39]. By contrast, angry rumination focuses attention on past threats (which may eventually become threatening in the future).

Future research could examine the extent to which the form of rumination that people adopt influences perceptions of distance. For instance, in depressive ruminators, concrete rumination (thinking about how one feels) can facilitate recovery from depression relative to abstract rumination (thinking about why one feels this way) [32]. Asking depressed people to concretely think about how they would solve social problems eliminates the normal impairment seen in depressed individuals [40]. By contrast, inducing an abstract mindset by asking depressed people to think about why they have the problem did not ameliorate this impairment. Interestingly, in Study 3, we found that the emotional events were recalled in a more abstract manner than the neutral events. This finding is noteworthy because abstract thinking is typically associated with increased psychological distance [4]. However, the present research along with the research on depressive rumination suggest that abstract rumination may not facilitate closure and paradoxically, may heighten psychological distress when thinking about sad events. By contrast, the evidence for angry rumination is more mixed, but seems to indicate that adopting a more abstract focus lessens anger and increases psychological distance [6, 41]. Thus, it is important to consider not only the type of emotional event, but also the unique effects of the construal level adopted during rumination. Understanding these subtle differences in rumination is important because frequent rumination is a risk factor for physiological stress reactivity and poor physical health [32, 42 – 43].

### Conclusions

This research contributes to the small, yet growing body of evidence showing that emotional processes influence perceptions of psychological distance [9 – 10]. Specifically, the present research identified rumination as a central factor in contributing to the feeling that negative events happened “just yesterday”. These findings suggest that expansion of construal level theory to “hot” cognitive-emotional processes may aid our understanding of psychological distance as well as generate new, testable hypotheses.

Reducing angry rumination may give people the distance they need to forgive others. Reducing guilty rumination may give people the distance they need to forgive themselves, and reducing depressive rumination may help people engage more effectively with life’s demands. Doing so might help improve the quality of life for people prone to rumination and those around them.

## Supporting Information

S1 Materials Modified Impact of Events Scale (IES) and table of zero-order correlations between the modified IES and key variables in Studies 2 and 3. ***p < .001.
(DOCX)

S1 Data Data from Studies 1–3. (ZIP)
