# Supplementary material for: Negative Emotional Events that People Ruminate about Feel Closer in Time
Source: PLoS One. 2015 Feb 25;10(2):e0117105. doi: 10.1371/journal.pone.0117105 (PMC4351980; doi:10.1371/journal.pone.0117105)
Supplement: S1 Materials — ***p < .001. (DOCX) [file pone.0117105.s001.docx]

**Materials S1**

Modified Impact of Events Scale (IES):

1. I thought about it when I didn’t mean to.
2. I had waves of strong feelings about it.
3. I had dreams about it.
4. I stayed away from reminders of it.
5. I felt as if it hadn’t happened or wasn’t real.
6. Other things kept making me think about it.
7. I was aware that I still had a lot of feelings about it, but I didn’t deal with them.
8. I tried not to think about it.

Zero-order correlations between the modified IES and key variables in Studies 2 and 3. ****p* < .001.

| Study 2 |  |
| --- | --- |
| Angry affect | .35*** |
| Guilty affect | .38*** |
| Emotional intensity | .48*** |
| Temporal distance | -.25*** |
| Study 3 |  |
| Angry affect | .37*** |
| Guilty affect | .33*** |
| Emotional intensity | .49*** |
| Temporal distance | -.41*** |
| Approach motivation | .15*** |
| Fluency | -.02 |
| Construal level | .27*** |
| Visual perspective | .02 |
| Vividness | .14*** |
